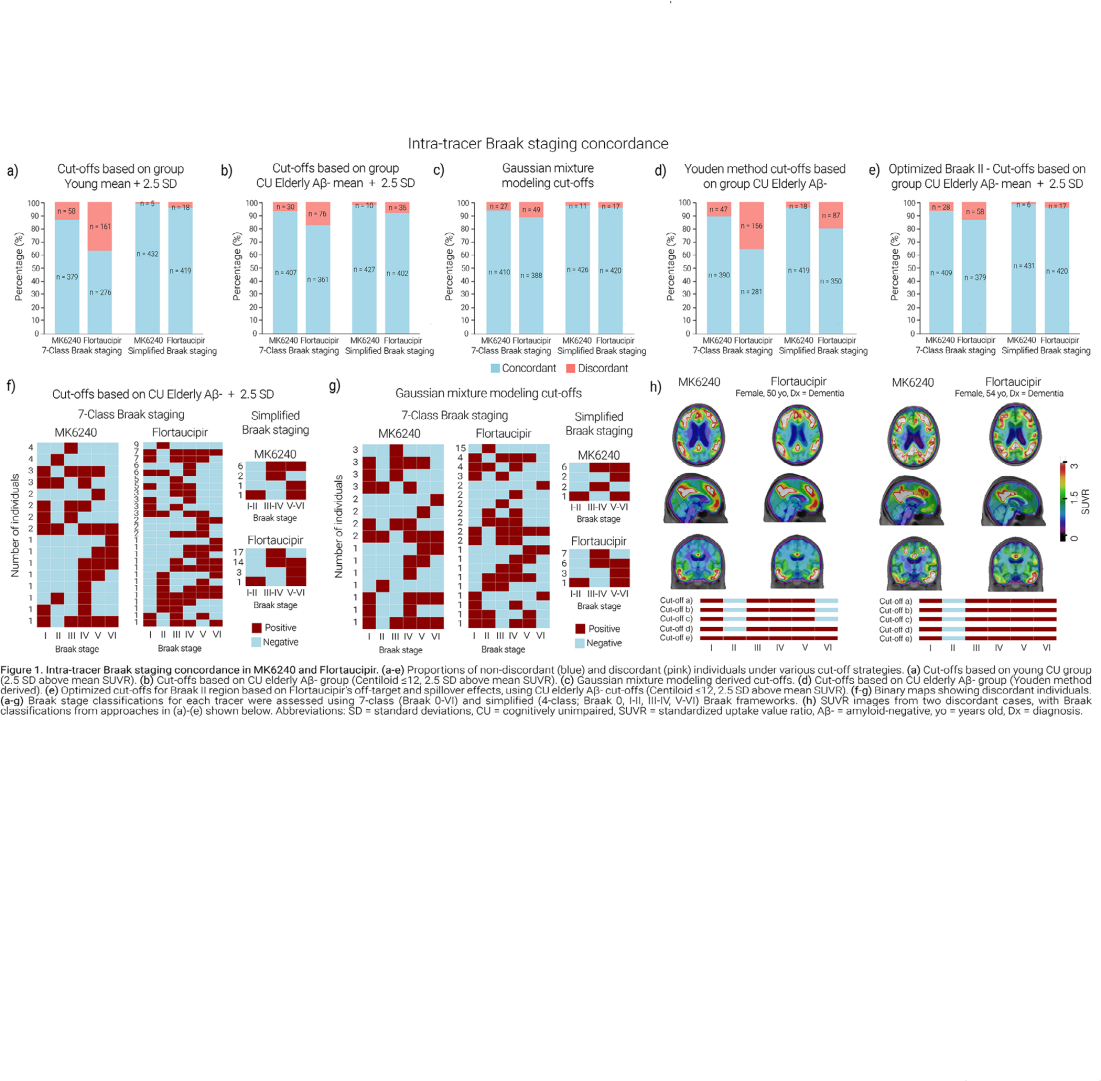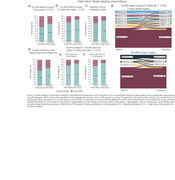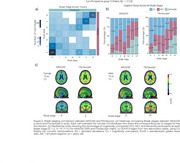# Head‐to‐head in vivo Braak staging with MK6240 and Flortaucipir

**DOI:** 10.1002/alz70862_110846

**Published:** 2025-12-23

**Authors:** Andreia Rocha, Bruna Bellaver, Emma Ruppert, Marina Scop Madeiros, Carolina Soares, Pamela C.L. Ferreira, Guilherme Povala, Livia Amaral, Guilherme Bauer‐Negrini, Firoza Z Lussier, Matheus Scarpatto Rodrigues, Joseph C. Masdeu, Dana L Tudorascu, David N. soleimani‐Meigooni, Juan Fortea, Val J Lowe, Hwamee Oh, Belen Pascual, Brian A. Gordon, Pedro Rosa‐Neto, Suzanne L. Baker, Tharick A Pascoal

**Affiliations:** ^1^ University of Pittsburgh, Pittsburgh, PA USA; ^2^ Houston Methodist Research Institute, Houston, TX USA; ^3^ University of California, San Francisco, San Francisco, CA USA; ^4^ Hospital de la Santa Creu i Sant Pau, Barcelona, Barcelona Spain; ^5^ Department of Radiology, Mayo Clinic, Rochester, MN USA; ^6^ Brown University, Providence, RI USA; ^7^ Washington University in St. Louis, St. Louis, MO USA; ^8^ McGill University, Montreal, QC Canada; ^9^ Lawrence Berkeley National Laboratory, Berkeley, CA USA

## Abstract

**Background:**

In vivo Braak staging stratifies patients across the AD spectrum and has the potential to harmonize tau PET tracer staging. This study aims to compare and test harmonization procedures for Braak staging individuals using MK6240 and Flortaucipir tau PET tracers.

**Methods:**

We assessed 437 participants across the AD spectrum (245 cognitively unimpaired (CU) and 192 cognitively impaired; mean age 68.5 ± 8.6) using head‐to‐head MK6240 and Flortaucipir scans. We computed SUVRs in Braak regions of interest (ROIs) and assessed four cut‐off methods for Braak positivity: (a) mean + 2.5 SD of young controls (age <28 years), (b) mean + 2.5 SD of elderly CU Aβ−, (c) Gaussian mixture modeling (GMM), and (d) the Youden index. Braak stages were assigned using seven (0 to VI) or four (0, I–II, III–IV, V–VI) categories. We evaluated inter‐ and intra‐tracer concordance (intra‐tracer, i.e., whether it follows the sequential Braak pattern).

**Results:**

The intra‐tracer seven‐class Braak staging concordance ranged from 63% to 94%. With the highest intra‐tracer Braak concordance being achieved when using GMM cutoffs: 94% (MK6240) and 89% (Flortaucipir; Figure 1). Inter‐tracer agreement concordance ranged from 56% to 76%. The highest concordance emerged from the CU Elderly Aβ– cutoff optimizing the Braak II region for spill‐off (Figure 2). Using the Braak staging simplified version improved intra‐tracer concordance in both tracers (MK6240as well as inter‐tracer agreement (86.5%). Most inter‐tracer discrepancies were observed at Braak stages II–IV. Despite showing staging discordances, the distribution of cognitive status across the Braak stages is similar for both tracers (Figure 3).

**Conclusion:**

These preliminary findings reveal some discrepancies in Braak staging when comparing MK6240 and Flortaucipir. Our results also suggest that adjustments in cutoffs and regions of interest can partially mitigate both inter‐ and intra‐tracer divergences. Finally, our analysis suggests robust concordance after adjustment and using 4 classes (0, I‐II, III‐IV, V‐VI).